# Design and Synthesis of AIE-Based Small-Molecule and Nanofibrous Film for Fluorescent Sensing Application

**DOI:** 10.3389/fchem.2021.727631

**Published:** 2021-08-06

**Authors:** Chunping Ma, Zhiyi Li, Chenglin Zhang, Gaoyi Xie, Yancheng Wu, Yangfan Zhang, Jinpeng Mo, Xi Liu, Ke Wang, Dong Xie, Yang Li

**Affiliations:** ^1^ Guangdong-Hong Kong Joint Laboratory for New Textile Materials, School of Textile Materials and Engineering, Wuyi University, Jiangmen, China; ^2^ School of Materials and Energy Engineering, Guizhou Institute of Technology, Guiyang, China; ^3^ School of Biotechnology and Health Sciences, Wuyi University, Jiangmen, China; ^4^ Institute of Bioengineering, Guangdong Academy of Sciences, Guangzhou, China

**Keywords:** fluorescent sensing, AIE, nanofibrous film, acid-induced discoloration, luminescence performance

## Abstract

Fluorescent sensors that respond to environmental conditions (temperature, pressure, and pH) have attracted widespread attention in recent years. Generally, traditional solid-state fluorescent materials tend to suffer from aggregation-induced quenching (ACQ) and difficulty of film forming, limiting their extensive applications. Therefore, researchers are focusing more and more attention on fluorescent sensors with aggregation-induced emission (AIE) effects. Herein, the article reports an AIE molecule (TPEBZMZ) containing tetraphenylethylene (TPE) and benzimidazole fragments. The fluorescence properties of TPEBZMZ in solution and aggregation states have been investigated, and the luminescence performance and aggregation structures of solid-state TPEBZMZ after force and acid treatments have been explored. The results show obvious AIE and fluorescent sensing properties of TPEBZMZ, presenting force- and acid-induced discolorations. Moreover, the TPEBZMZ-based fluorescent nanofibrous film is fabricated by electrospinning the solution of TPEBZMZ blended with polylactic acid (PLA), which shows a good nanofiber film structure and exhibits reversible acid-induced discoloration property, even with only 0.5 wt% TPEBZMZ. This work provides a simple strategy to achieve stimulus-responsive fluorescent film.

## Introduction

Fluorescent films composed of π-conjugated materials-based fluorescent sensors and membranous materials are promising sensor technology due to their real-time detection, easy fabrication, non-invasiveness detection, and mechanical stability and flexibility (Guan et al., 2015; Miao et al., 2016). In recent years, they have been widely used in detecting residual pesticides (Peng et al., 2021), enzymes (Zhao et al., 2017a; Zhao et al., 2017b), pollutants (Lee et al., 2019; Zhao et al., 2020b; Sun et al., 2021), explosives (Zhou et al., 2014; Sun et al., 2015; Li et al., 2018; Hao et al., 2021), metal ions (Chen et al., 2020), gases (Moscoso et al., 2020), and temperature and humidity (Jiang et al., 2020; Li et al., 2020), which greatly expanded the applications of fluorescence sensors. Generally, high selectivity and sensitivity are the key characteristics of a successful membranous fluorescent sensor (Sun et al., 2015). Selectivity is mainly related to the specific interaction between fluorescent probe and environmental stimulus or detection objects (Zhou et al., 2017), showing differentiated luminescence characteristics, while sensitivity is primarily determined by fluorescence intensity (Song et al., 2015). Traditional fluorescent materials usually present weak fluorescence in the solid state but high fluorescence in solution due to the widely known aggregation-caused quenching (ACQ) effect. ACQ greatly limits the application of traditional fluorescence materials (Hong et al., 2011). Aggregation-induced emission (AIE), proposed by Tang’s research team in 2001, can be used as a perfect solution to the ACQ issue (Luo et al., 2001). AIE behavior is just the opposite of the ACQ fluorescence behavior of traditional fluorescent compounds: AIE luminogen (AIEgen) emits weak fluorescence in solution, whereas it emits strong fluorescence when aggregated or in a solid state (Luo et al., 2001). The AIE phenomenon is related to intermolecular rotation, restricting these rotations in solid state, inducing the fluorescent emission of the corresponding compound (Qin et al., 2012; Liu et al., 2019; Zhao et al., 2020a). Therefore, AIEgen-based films will show specifically emissive characteristics. Thus, AIEgen-based fluorescent films will provide an effective strategy for the development of fluorescent film sensors.

Electrospinning is an effective method to obtain polymer-based nanofibrous films, compared with casting films, showing a larger specific surface area and higher porosity (Xue et al., 2019). Thus, fluorescent films fabricated by electrospinning can improve the sensitivity and response rate (Zhao et al., 2017b). Therefore, much attention has been devoted to nanofibrous fluorescent films to explore their specific sensing properties. For example, Li et al. (2019) have developed an epoxy resin system functionalized with the typical AIE tetraphenylethylene (TPE) groups, forming nanofibrous films by electrospinning, and finally obtained temperature-sensitive fluorescent films probes. Yang et al. (Yang et al., 2018) have reported specific composite nanofibrous films by electrospinning of AIE small molecules (oME-TPA) and polyvinyl alcohol (PVA) and further studied its piezochromic properties related to sensitivities. Zhao et al. (2017a) have grafted TPE derivatives and phloxine B onto electrospun nanofiber film, inducing the static quenching of phloxine B through protamine adsorption and combining them with the aggregation of TPE-based groups, promoting fluorescence emission of nanofiber, which realized effective monitoring of different concentrations of heparin. Therefore, the design and development of novel AIE molecules containing nanofiber fluorescent films will benefit the realization of efficient fluorescent sensing applications.

Herein, we report a novel compound (TPEBZMZ) containing the classic AIE group tetraphenylethylene (TPE) and benzimidazole unit. The TPE group renders the TPEBZMZ molecule AIE property, and the structure of the benzimidazole unit will offer the potential of acid-induced discoloration. The relationship between the structure, aggregate, and luminescence properties of TPEBZMZ in different states and force and acid treatments has been systematically studied. Specifically, TPEBZMZ shows an obvious AIE phenomenon and fluorescent sensing properties of force- and acid-induced discolorations. More importantly, we prepared TPEBZMZ-based fluorescent nanofibrous film by electrospinning the solution of TPEBZMZ blended with polylactic acid (PLA), and the nanofibrous film displays a good reversible acid-induced discoloration property. Significantly, this work provides a simple strategy to prepare fluorescent sensing film.

## Materials and Methods

### Materials

1-Bromo-1,2,2-triphenylethene (P_3_Br) was purchased from Macklin Biochemical Co., Ltd. (Shanghai, China). 4-Formylphenylboronic acid, tetrabutylammonium bromide (TBAB), tetrakis(triphenylphosphine) palladium [Pd(PPh_3_)_4_], 2-(cyanomethyl) benzimidazole, and tetrabutylammonium hydroxide (TBAH) were purchased from Energy Chemical. Polylactic acid (PLA, 4032D) was purchased from NarureWorks (America). All of the above reagents were used as received. Potassium carbonate (K_2_CO_3_), potassium hydroxide (KOH), tetrahydrofuran (THF), ethanol, and dichloromethane (DCM) were purchased the analytical grade from Guangzhou Chemical Reagent Factory (Guangzhou, China) and used without further purification.

### Synthesis of P_4_A and (E)-2-(1H-Benzo[d]imidazol-2-yl)-3-(4-(1,2,2-triphenylvinyl)phenyl) Acrylonitrile (TPEBZMZ)

The intermediate of P_4_A was synthesized according to a previous literature method (Xu et al., 2013).

P_4_A (1.080 g, 3 mmol), 2-(cyanomethyl) benzimidazole (0.471 g, 3 mmol), and ethanol (20 ml) were mixed and stirred in a 50 ml two-necked round-bottomed flask at room temperature. After adding tetrabutylammonium hydroxide (TBAH, 0.8 M, 10 drops) dropwise, the mixture was stirred continually at room temperature for 4 h. The precipitate was filtrated and washed three times using ethanol, resulting in a yellow powder (1.087 g, yield 72.6%). ^1^H NMR (500 MHz, CDCl_3_) *δ* (ppm): 9.58 (s, 1 H), 8.41 (s, 1 H), 7.77 (d, 2 H, J = 8.32 Hz), 7.34 (dd, 2 H, J = 3.12, 6.09 Hz), 6.01–7.23 (m, 19 H). ^13^C NMR (125 MHz, CDCl_3_) *δ* (ppm): 148.35, 146.46, 146.34, 143.19, 143.09, 143.01, 139.81, 132.19, 131.37, 131.33, 131.29, 130.55, 129.61, 127.99, 127.95, 127.71, 127.12, 126.86, 117.01, 98.63. FT-IR (KBr): 2924, 2854, 1593, 1491, 1443, 747, 700, 616. ESI-MS, m/z: 500.21 ([M + H+], calcd for C36H26N3, 500.20).

### Characterization

^1^H NMR and ^13^C NMR spectra were recorded on a Bruker AVANCE NEO 500 spectrometer. Mass spectra were measured on an LTQ Orbitrap LCMS spectrometer. FT-IR spectra were recorded using Nicolet iS5 spectrometer (KBr pellet). UV–visible absorption spectra (UV–vis) were obtained via a SHIMADZU UV-2700 spectrophotometer. Fluorescence spectra (PL) were determined by a Shimadzu RF-6000 spectrometer with a slit width of 3 nm for excitation and 5 nm for emission. Thermal behaviors were studied using differential scanning calorimetry (DSC) on a NETZSCH thermal analyzer (DSC214Polyma) with both heating and cooling rates of 20°C/min under a N_2_ atmosphere. Powder X-ray diffraction (PXRD) measurements were carried out at 298 K on a Rigaku X-ray diffractometer (Ultima IV, Japan) with an X-ray source of Cu Kα (*λ* = 0.1541 nm) at 40 kV and 40 mA, at a scan rate of 5°(2θ)/min from 5° to 60°. The frontier molecular orbital distributions of TPEBZMZ and acid fumed TPEBZMZ (TPEBZMZ-HCl) were determined using density functional theory (DFT) using Gaussian 09 at the B3LYP/6-311G(d,p) level. After 1 min of gold coating, the surface morphologies of nanofibers were observed by a scanning electron microscope (Sigma 500, Zeiss, German) equipped with an in-lens detector at 15 kV. Fluorescent microscopic images were recorded on a laser confocal microscope (Leica DMI8, Germen) in an XY scan mode, using an argon ion laser source with a wavelength of 488 nm.

### Electrospinning Process of PLA-TPEBZMZ and PLA Nanofibrous Films

The electrospinning process was performed on an E05 electrospinning apparatus (supplied by Lepton Technology Co., Ltd., Foshan, China), using a 10 wt% PLA/(DMF:DCM = 1:2) solution with or without 0.05 wt% TPEBZMZ as a spinning solution. The nanofibers were collected by a rotating metal cylinder (100 r/min) covered with release paper, with an immobile distance of 20 cm away from the needle tip of the spinneret (0.7 mm inner diameter). The spinning voltage and fluid flow rate were set to 12 kV and 2 ml/h, respectively. The obtained nanofibrous films were dried in a vacuum oven at 70°C for another 12 h to remove the residual solvent completely.

## Result and Discussion

### Synthesis and Characterization

**SCHEME 1:**
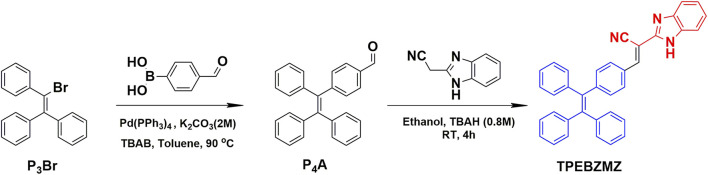
Synthetic route and chemical structure of TPEBZMZ.

The synthetic route of TPEBZMZ is shown in Scheme 1. The intermediate molecule P_4_A was acquired *via* Suzuki coupling from P_3_Br and 4-formylphenylboronic acid. The final product TPEBZMZ was obtained by Knoevenagel reaction with P_4_A and 2-(cyanomethyl) benzimidazole. As shown in Supplementary Figures 1–4, TPEBZMZ was characterized using ^1^H NMR and ^13^C NMR spectroscopies, mass spectrometry, and Fourier transform infrared spectroscopy, confirming that the target compound TPEBZMZ has been successfully synthesized. The thermal stability of TPEBZMZ was investigated by thermal gravity analysis (TGA) under a nitrogen atmosphere, and the result is presented in Supplementary Figure 5. TPEBZMZ showed good thermal stability with 5% weight loss at a temperature (*T*_d_) of 366°C.

### AIE Property of TPEBZMZ

**FIGURE 1:**
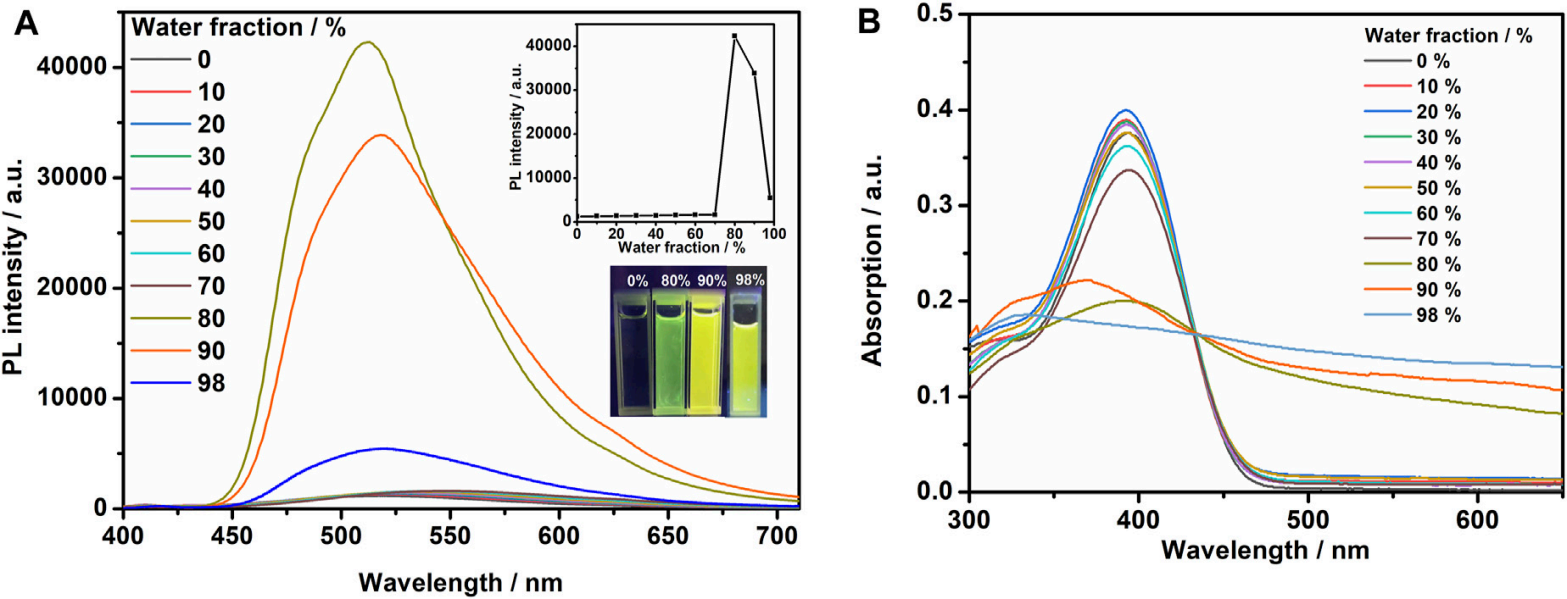
**(A)** Fluorescence spectra of TPEBZMZ in THF/water mixed solvents (10 mM) with different water fractions. The insets show changes in peak intensity of the fluorescence spectra and emission images of the compounds in different water fraction mixtures under 365 nm UV illumination. **(B)** UV–vis absorption spectra of TPEBZMZ in THF/water mixed solvents (10 mM) with different water fractions.

Due to the state-of-the-art AIE functional unit, TPE, we proposed that the TPEBZMZ molecule can exhibit the AIE effect, which was firstly examined by the photoluminescence (PL) fluorescence emission spectra of TPEBZMZ/THF/H_2_O solutions with different water content. The resulting spectra, the plot of PL intensity vs. water fraction, and the fluorescence photo images of specific water fraction are shown in Figure 1A. The fluorescence emission of the TPEBZMZ/THF solution in the presence of only the good solvent THF is very weak, and the fluorescence of the solution is almost invisible to the naked eye. When the water content is lower than 70%, the fluorescence spectra show few changes. In contrast, the fluorescence intensity increases sharply, when the water content reaches 80%, and the fluorescence intensity is about 36 times compared to 0% water content. The fluorescence intensity begins to decrease after the water content is further increased to 90%, within a sharp drop of fluorescence intensity at 98% of water content. We speculate that the reason for the change in fluorescence intensity is because TPEBZMZ aggregation started with 80% of water content, and the size of aggregates increased gradually when the water fraction increased to 98%, leading to a remarkable light scattering phenomenon (Thomas et al., 2007). Thus, the PL fluorescence experiment preliminarily confirmed the AIE property of TPEBZMZ.

Optical absorption properties of TPEBZMZ/THF/H_2_O solutions with different water content were investigated using UV–vis absorption spectroscopy. As shown in Figure 1B, the solution absorption spectra exhibit similar characteristics under 0–70% water content, while the obvious decreases of absorption intensities were demonstrated in >80% water content solutions. Moreover, the spectra of >80% water content show the obvious tailing phenomenon in absorption curves, especially presented in the 98% water content solution, which can be attributed to the Mie scattering effect of nano aggregates (Zhang et al., 2015). The tendency of absorption spectra is consistent with the results of fluorescence emission spectra of TPEBZMZ/THF/H_2_O mixture solution with different water content. These results further confirmed that TPEBZMZ shows the AIE effect, which will benefit its further fluorescent sensor application.

### Mechanochromism Property of TPEBZMZ

**FIGURE 2:**
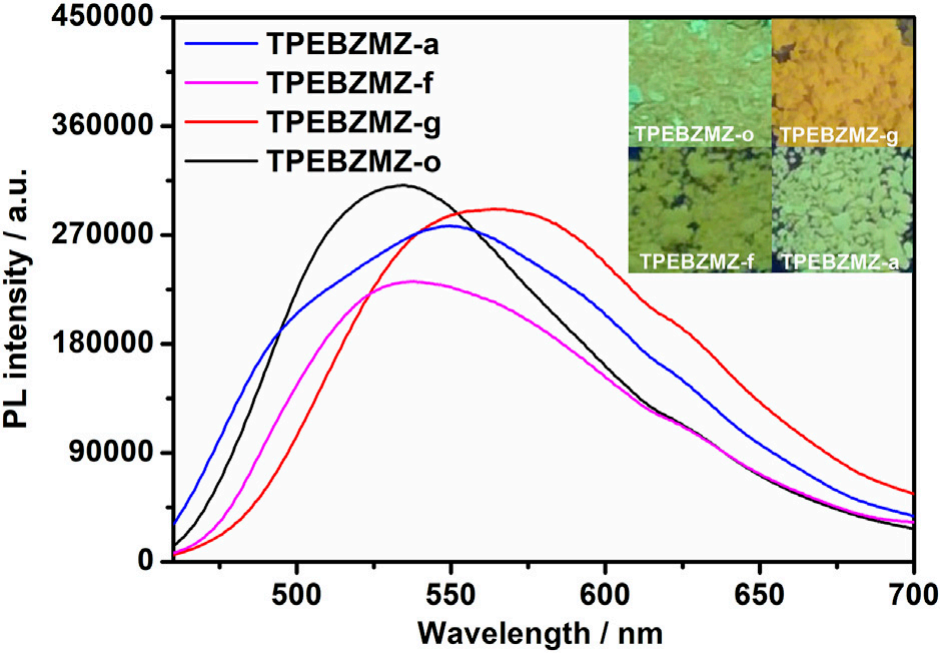
Fluorescence spectra and images of TPEBZMZ: TPEBZMZ-o, the original powder; TPEBZMZ-g, ground powder from TPEBZMZ-o; TPEBZMZ-f, CH_2_Cl_2_-fumed (2 h) powder from TPEBZMZ-g; TPEBZMZ-a, annealed (240°C, 1 h) powder from TPEBZMZ-g.

To evaluate the stimulus-response characteristic of TPEBZMZ, we first tested the fluorescence performance of the original TPEBZMZ (TPEBZMZ-o) powder. As shown in Figure 2, TPEBZMZ-o shows a strong yellow-green fluorescence emission with a PL peak at 534 nm. The ground powder (TPEBZMZ-g) was obtained by grinding TPEBZMZ-o, which releases an orange-yellow fluorescence emission with a PL peak of 565 nm. The 31 nm red shift of fluorescence peaks of TPEBZMZ-o and TPEBZMZ-g demonstrated that the preliminary mechanochromism property of TPEBZMZ. The fluorescence color returned to yellow-green when the TPEBZMZ-g sample was treated with dichloromethane (DCM) fumigation (TPEBZMZ-f), and TPEBZMZ-f shows a blue-shift fluorescence spectrum, showing a similar wavelength range of TPEBZMZ-o. The annealed sample (TPEBZMZ-a) of TPEBZMZ-g also exhibits a similar effect with a blue-shift of fluorescence spectrum. Moreover, the results of the cycling measurement (Supplementary Figure 6) indicate that TPEBZMZ displays good reversible mechanochromism properties.

**FIGURE 3:**
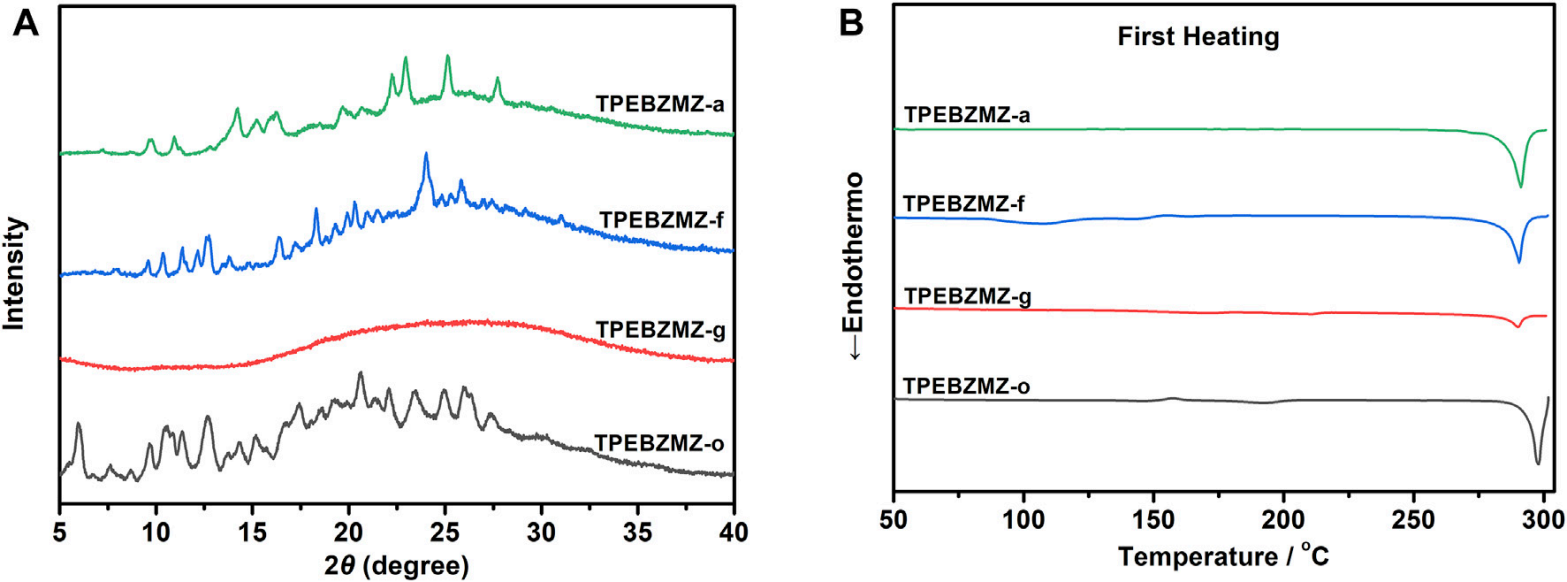
**(A)** The PXRD patterns of TPEBZMZ; **(B)** DSC curves of TPEBZMZ.

Solid-state TPEBZMZ changes from yellow-green to orange-yellow after being mechanically stimulated. We speculate that the reason is the change in the aggregation state of the TPEBZMZ molecules due to the action of external mechanical forces (Ma et al., 2016; Ma et al., 2017). Accordingly, the aggregate structures of the TPEBZMZ powder with different treatments were investigated by powder X-ray diffraction (PXRD) measurements. As shown in Figure 3A, a large number of sharp diffraction peaks appeared in the PXRD curve of TPEBZMZ-o, suggesting a relatively regular molecular arrangement and good crystallinity presented in the initial state of TPEBZMZ. While the ground sample TPEBZMZ-g shows weak and even disappeared diffraction peaks, it indicates a relatively disordered and amorphous state exhibited in the TPEBZMZ-g sample. Moreover, the diffraction peaks are partially restored while TPEBZMZ-g is annealed, and the major peaks of the fumed TPEBZMZ-f are also restored and similar to those of TPEBZMZ-o. These results demonstrated the varied aggregated structures of TPEBZMZ with different treatments, which is consistent with its mechanochromism property.

Aggregate structures of TPEBZMZ with different treatments were further characterized by differential scanning calorimetry (DSC) measurements. As shown in Figure 3B, in the first heating curves, TPEBZMZ-o shows an obvious melting peak at 298°C (enthalpy of 25.80 J/g), while TPEBZMZ-g displays a weak melting peak at 290°C with an enthalpy of 4.63 J/g, suggesting that the mainly amorphous state is presented in the TPEBZMZ-g sample. TPEBZMZ-f and TPEBZMZ-a also have obvious crystal melting peaks near 290°C, but the melting enthalpies (23.77 J/g and 25.90 J/g, respectively) are slightly lower than or almost the same as TPEBZMZ-o, indicating that a certain rearrangement of amorphous structure of the TPEBZMZ sample appeared under annealing or DCM fumigation treatments.

### Acid-Base Discoloration Property of TPEBZMZ

**FIGURE 4:**
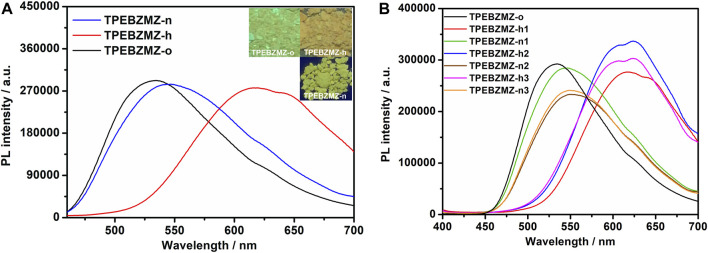
**(A)** Fluorescence spectra and images of TPEBZMZ: TPEBZMZ-o, the original powder; TPEBZMZ-h, HCl-fumed (10 min) powder from TPEBZMZ-o (HCl gas was released from a 37% concentrated HCl aqueous solution); TPEBZMZ-n, NH_3_-fumed (15 min) powder from TPEBZMZ-o (NH_3_ gas was released from a 25% concentrated NH_3_ aqueous solution). **(B)** Repeated fluorescence switch of TPEBZMZ by HCl fuming and NH_3_ fuming cycles.

The protonateable benzimidazole unit presented in the TPEBZMZ molecule renders the variable electron cloud density of TPEBZMZ after being protonated, further, in turn, affecting its fluorescence performance. Thus, TPEBZMZ-o was fumigated by hydrogen chloride gas to obtain the protonated acid-fumigated TPEBZMZ powder (TPEBZMZ-h). As shown in Figure 4A, the TPEBZMZ-h sample displays orange fluorescence with an emission peak at 589 nm, which shows an obvious red shift of 55 nm compared to the TPEBZMZ-o solid. Furthermore, the TPEBZMZ-h solid fumigated NH_3_ vapor (TPEBZMZ-n) shows a fluorescence color turning back to yellow-green, and its fluorescence spectrum is almost restored to that of TPEBZMZ-o. Figure 4B shows the repeated fluorescence spectra of TPEBZMZ by HCl fuming and NH_3_ fuming cycles, displaying modest repeatability of acid-base discoloration under three cycles. These results prove that the TPEBZMZ powder exhibits good reversible acid-base discoloration performance.

**FIGURE 5:**
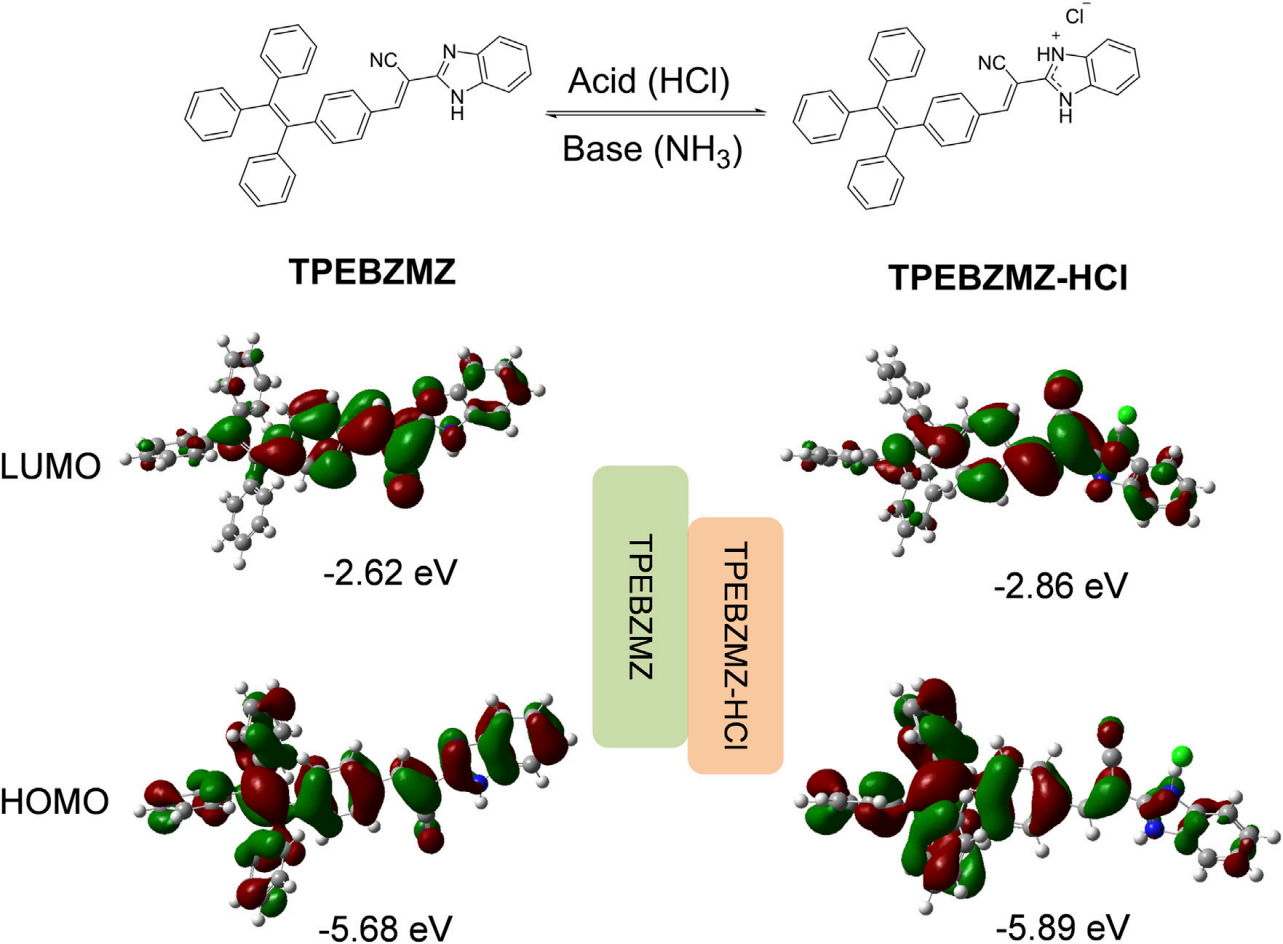
Proposed reaction mechanism for TPEBZMZ treated with HCl and NH_3_, and the frontier molecular orbital distributions of TPEBZMZ and HCl-fumed TPEBZMZ (TPEBZMZ-HCl) determined using DFT by Gaussian 09 at the B3LYP/6-311G(d,p) level.

The proposed reaction mechanism about TPEBZMZ treated with HCl and NH_3_ is shown in Figure 5, and the frontier molecular orbital distributions of TPEBZMZ and HCl-protonated TPEBZMZ (TPEBZMZ-HCl) were further determined using DFT by Gaussian 09 at the B3LYP/6-311G(d,p) level. The electron density distribution of the lowest unoccupied molecular orbital (LUMO) of TPEBZMZ is localized in the central molecule and tends to be fastened to the cyano unit. The highest occupied molecular orbital (HOMO) of TPEBZMZ shows a well-proportioned electron density distribution. Moreover, the electron density distributions of the LUMO and HOMO of TPEBZMZ-HCl exhibit a delocalization effect, which tends to localize in the benzimidazole hydrochloride unit and TPE unit, respectively. Correspondingly, TPEBZMZ and TPEBZMZ-HCl exhibit LUMO/HOMO levels of −2.62/−5.68 and −2.86/−5.89 eV, respectively, presenting the bandgaps of 3.06 and 3.03 eV. The red-shift fluorescence spectra of HCl-protonated TPEBZMZ could be attributed to the delocalization effect of its electron density distributions, which may explain the acid-base discoloration property of TPEBZMZ.

### Fluorescent Sensing Property of TPEBZMZ-Based Nanofibrous Film

**FIGURE 6:**
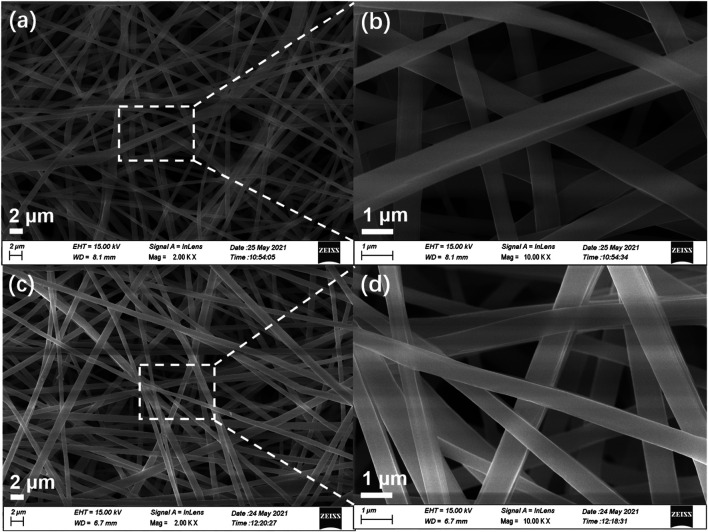
SEM images of **(A,B)** PLA nanofibrous film and **(C,D)** TPEBZMZ-m. **(A,C)** Large scale; **(B,D)** high resolution.

Generally, small-molecule fluorescent powders exhibit poor film-forming properties requiring expensive film-forming equipment (e.g., vacuum evaporator), which will limit their practical application (Hong et al., 2011). To overcome this drawback, TPEBZMZ was blended with a PLA dimethylformamide (DMF)/DCM mix solution by electrospinning to obtain the TPEBZMZ-based nanofibrous film (TPEBZMZ-m). The morphology of TPEBZMZ-m and a pure PLA nanofibrous film was tested using scanning electron microscopy (SEM). The corresponding SEM images are shown in Figures 6A–D. TPEBZMZ-m shows an almost similar microstructure compared to that of the pure PLA nanofibrous film, both of which possess filaments with diameters of 500–800 nm.

**FIGURE 7:**
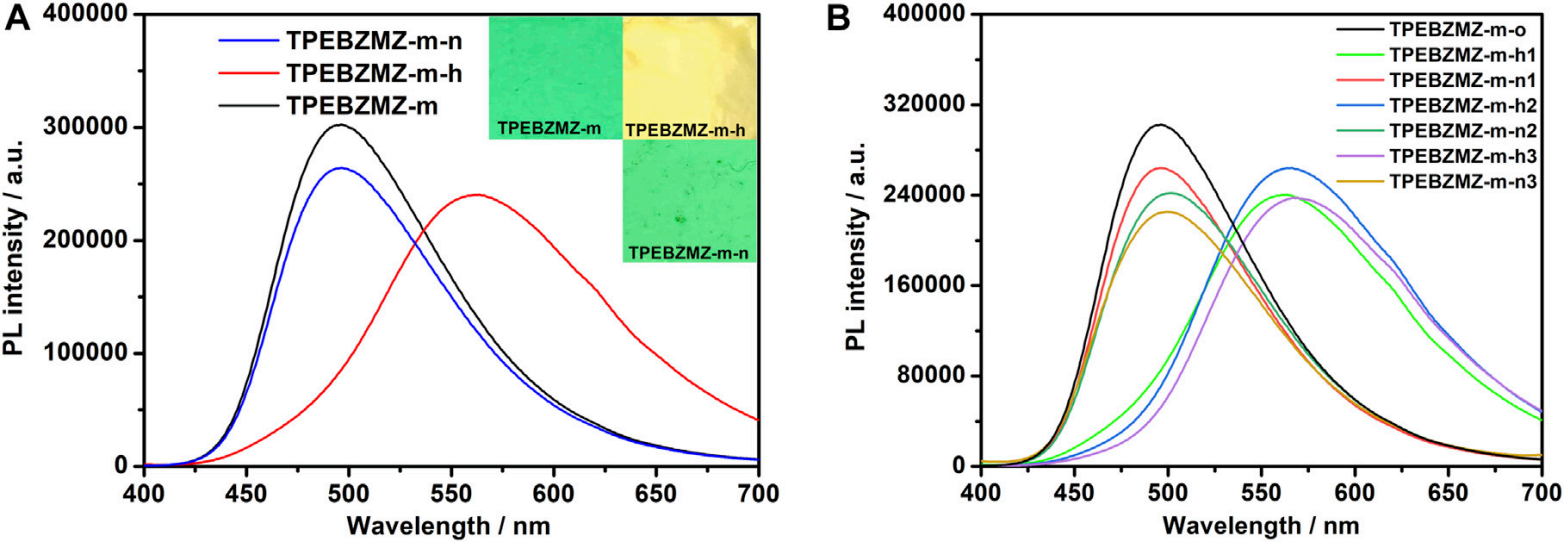
**(A)** Fluorescence spectra and images of TPEBZMZ-m: TPEBZMZ-m, the original TPEBZMZ-based nanofibrous film; TPEBZMZ-m-h, HCl-fumed (24 h) film from TPEBZMZ-m (HCl gas was released from a 37% concentrated HCl aqueous solution); TPEBZMZ-m-n, NH_3_-fumed (15 min) film from TPEBZMZ-m-h (NH_3_ gas was released from a 25% concentrated NH_3_ aqueous solution). **(B)** Repeated fluorescence switch of TPEBZMZ-m by HCl fuming and NH_3_ fuming cycles.

To evaluate the fluorescent sensing property of TPEBZMZ-m, the acid-induced discoloration experiment was performed. The results of the acid-induced discoloration experiment are shown in Figure 7A. Unlike the pure PLA nanofibrous film (Supplementary Figure 7B), the nascent TPEBZMZ-m film emits significant green fluorescence under ultraviolet light (confirmed by laser confocal microscope, Supplementary Figures 7C–E), even with only 0.5 wt% TPEBZMZ. After fuming by HCl vapor, the resulting film (TPEBZMZ-m-h) exhibits an obvious yellow fluorescence color. Moreover, when the TPEBZMZ-m-h sample was fumed with a NH_3_ vapor (TPEBZMZ-m-n), the fluorescence of the sample became green again. To quantitatively characterize the fluorescence emission wavelength of TPEBZMZ-m after different treatments, we used fluorescence spectroscopy to test the electrospinning nanofibrous film samples after different treatments. As shown in Figure 7A, the TPEBZMZ-m sample shows a fluorescence emission peak of 496 nm, while the TPEBZMZ-m-h sample exhibits a fluorescence emission peak of 563 nm, with a red shift of 67 nm compared to TPEBZMZ-m. Meanwhile, the TPEBZMZ-m-n sample shows an obvious blue-shift fluorescence emission compared to the TPEBZMZ-m-h sample and presenting a fluorescence emission peak at 496 nm, coinciding with the spectroscopy of TPEBZMZ-m. Figure 7B shows the repeated fluorescence spectra of TPEBZMZ-m by HCl fuming and NH_3_ fuming cycles, displaying good repeatability of acid-base discoloration under three cycles. The well acid-base discoloration repeatability of TPEBZMZ-based nanofibrous film suggests its good potential on fluorescent film sensors application. These results demonstrated that electrospinning acts as a simple and efficient method to prepare TPEBZMZ-based nanofibrous fluorescent film with good reversible acid-induced discoloration property, which provides an effective strategy for developing fluorescent film sensors.

## Conclusion

In conclusion, we have developed an AIE molecule (TPEBZMZ) containing tetraphenylethylene (TPE) and benzimidazole fragments, which is synthesized from 2-cyanomethylbenzimidazole and 4-(1,2,2-tristyryl)benzaldehyde. TPEBZMZ powder shows obvious AIE and fluorescent sensing properties and exhibits force- and acid-induced discoloration phenomena. More importantly, the TPEBZMZ-based fluorescent nanofibrous film is fabricated by electrospinning the solution of TPEBZMZ blended with polylactic acid (PLA), exhibiting a good nanofiber film structure and well reversible acid-induced discoloration properties. This work demonstrates a simple strategy to achieve stimulus-responsive fluorescent film, which will benefit the development of fluorescent film sensors.

## Data Availability

The original contributions presented in the study are included in the article/Supplementary Material; further inquiries can be directed to the corresponding author/s.
