# Supplementary material for: Design and Synthesis of AIE-Based Small-Molecule and Nanofibrous Film for Fluorescent Sensing Application
Source: Front Chem. 2021 Aug 6;9:727631. doi: 10.3389/fchem.2021.727631 (PMC8377474; doi:10.3389/fchem.2021.727631)
Supplement: Supplementary file 1 [file DataSheet1.DOCX]

SUPPLEMENTARY INFORMATION FOR

**Design and Synthesis of AIE-based Small Molecule and Nanofibrous Film for Fluorescent Sensing Application**

**Chunping Ma^1,2^, Zhiyi Li^1^, Chenglin Zhang^3^, Gaoyi Xie^3^*, Yancheng Wu^1^, Yangfan Zhang^1^, Jinpeng Mo^1^, Xi Liu^1^*, Ke Wang^4^, Dong Xie^4^ and Yang Li^2^***

^1^Guangdong-Hong Kong Joint Laboratory for New Textile Materials, School of Textile Materials and Engineering, Wuyi University, Jiangmen 529020, China

^2^School of Materials and Energy Engineering, Guizhou Institute of Technology, Guiyang, 550003, China

^3^School of Biotechnology and Health Sciences, Wuyi University, Jiangmen 529020, China

^4^Institute of Bioengineering, Guangdong Academy of Sciences, Guangzhou, 510316, China


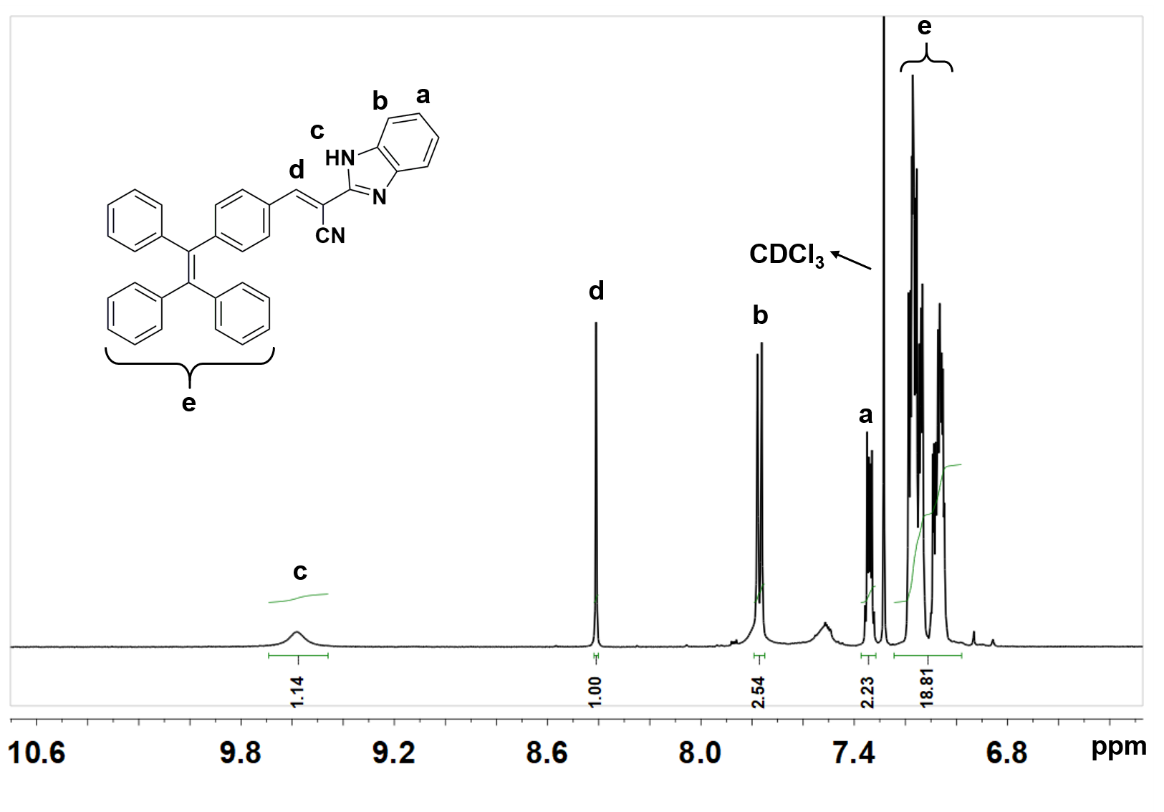


Figure S1 ^1^H-NMR spectrum of TPEBZMZ


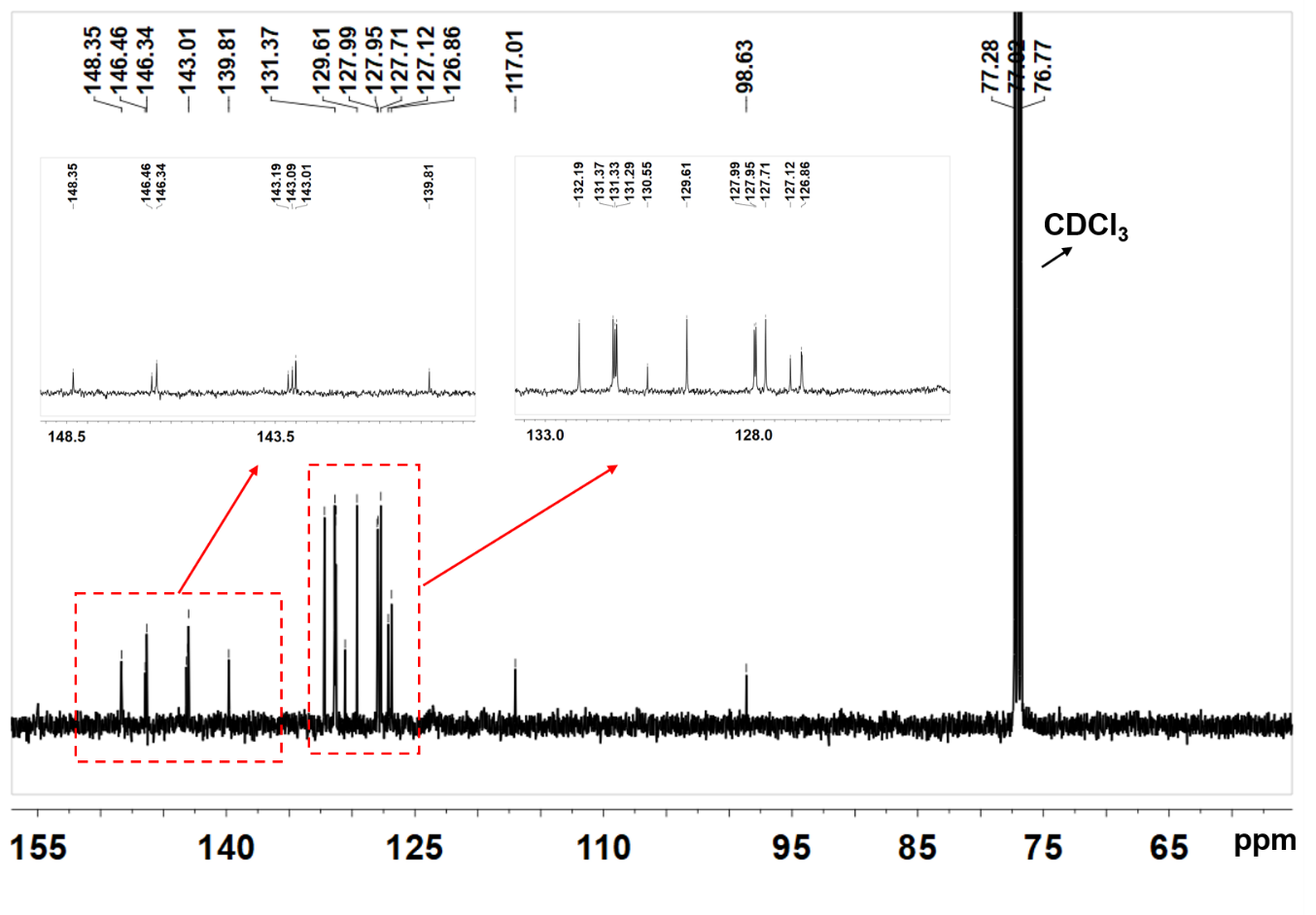


Figure S2 ^13^C-NMR spectrum of TPEBZMZ


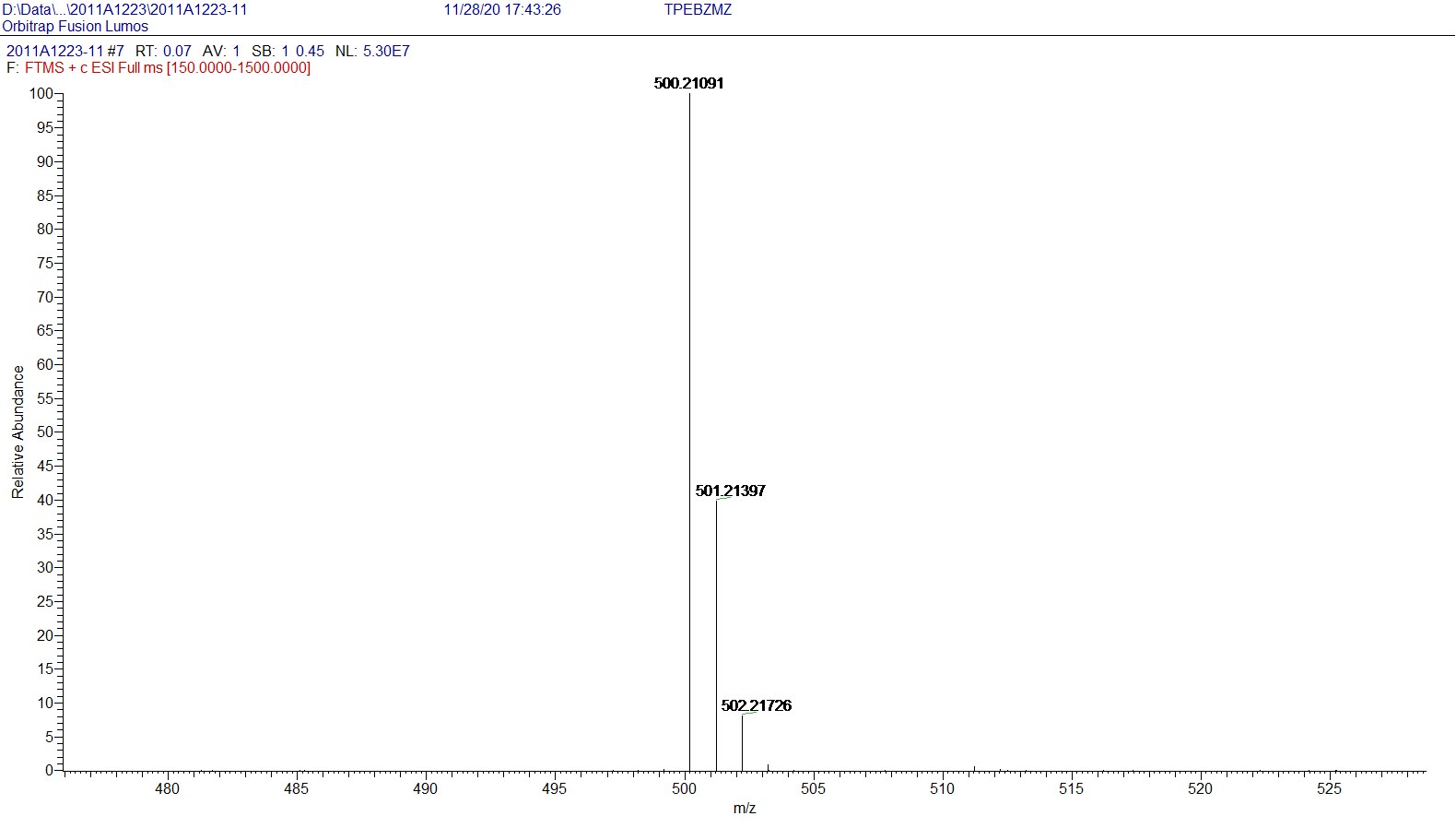


Figure S3 MS spectrum of TPEBZMZ





Figure S4 FT-IR spectrum of TPEBZMZ





Figure S5 TGA curve of TPEBZMZ







Figure S6 Fluorescence spectra of TPEBZMZ under different treatments. (o) the original powder, (g) treated by griding; (a) treated by annealing; (f) treated by CH_2_Cl_2_ fuming.


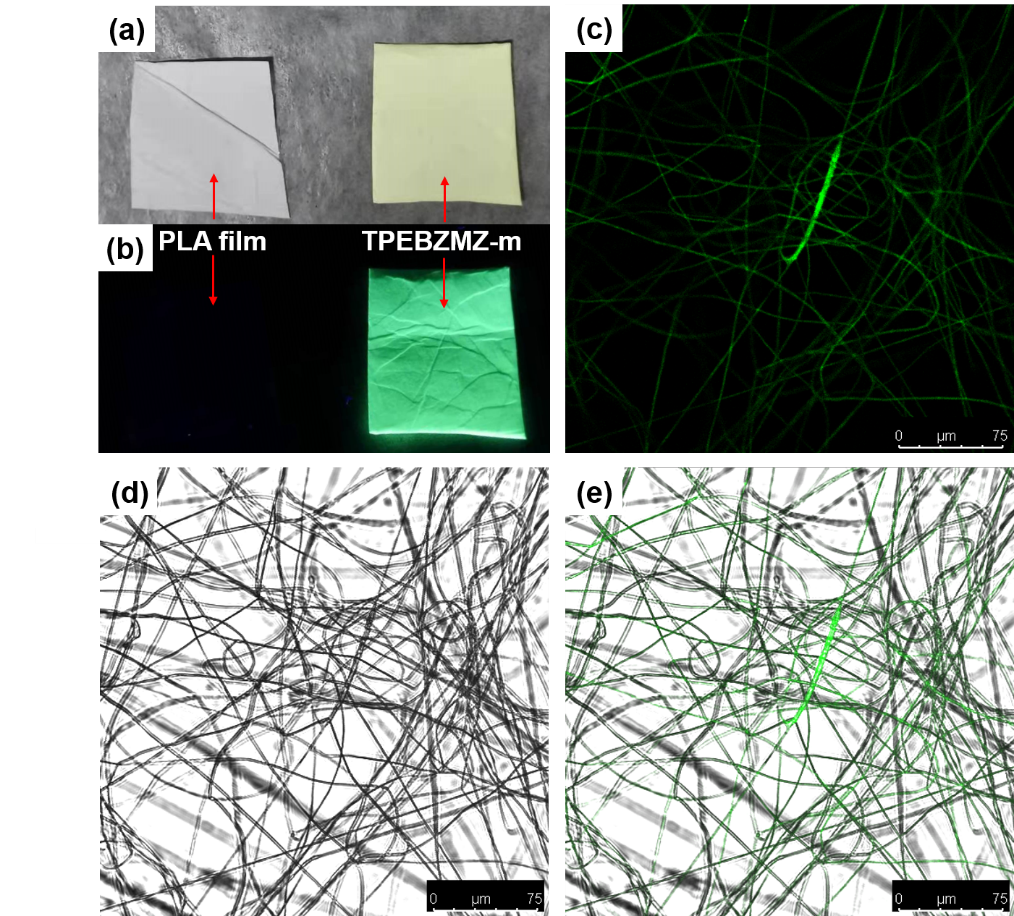


Figure S7 (a, b) Images of pure PLA nanofibrous film and TPEBZMZ-m. (a) under visible light illumination; (b) under 365 nm UV illumination; (c-e) Microscopic images of TPEBZMZ-m. (c) excited with 488 nm laser (d) bright field; (e) merged image of c and d.
